# Favorable outcome of immunotherapy in a rare subtype of hepatocellular carcinoma: a case report and literature review

**DOI:** 10.3389/fonc.2024.1358804

**Published:** 2024-04-23

**Authors:** Anita Archwamety, Nique Kunapinun, Sirinart Sirinvaravong, Piyaporn Apisarnthanarak, Charuwan Akewanlop, Krittiya Korphaisarn

**Affiliations:** ^1^ Division of Medical Oncology, Department of Medicine, Faculty of Medicine Siriraj Hospital, Mahidol University, Bangkok, Thailand; ^2^ Department of Pathology, Faculty of Medicine Siriraj Hospital, Mahidol University, Bangkok, Thailand; ^3^ Division of Endocrinology and Metabolism, Department of Medicine, Faculty of Medicine Siriraj Hospital, Mahidol University, Bangkok, Thailand; ^4^ Department of Radiology, Faculty of Medicine Siriraj Hospital, Mahidol University, Mahidol University, Bangkok, Thailand

**Keywords:** scirrhous HCC, scirrhous hepatocellular carcinoma, immune checkpoint inhibitor, immune related adverse event (irAE), hepatocellular carcinoma (HCC), type 1 DM, atezolizumab, bevacizumab

## Abstract

Scirrhous hepatocellular carcinoma (S-HCC) represents an uncommon subtype of HCC. During radiological evaluation this unique subtype is frequently mistaken as cholangiocarcinoma, fibrolamellar HCC, or metastatic adenocarcinoma. Here, we present the case of a 50-year-old woman with a large hepatic mass. A triple-phase computed tomography of the liver revealed an arterial enhancing lesion without portovenous washout at hepatic segment 4a/8. The liver biopsy showed hepatocellular characteristics and was positive for Hep Par 1, CK7, CK19, Arginase 1 and CEA, indicating atypical S-HCC. This patient had achieved tumor control with combined treatment with atezolizumab plus bevacizumab and was then treated with lenvatinib after tumor progression. The patient died 15 months after the initial diagnosis.

## Introduction

Scirrhous hepatocellular carcinoma (S-HCC) is an uncommon subtype of HCC, representing approximately 4.6% of all cases (1), which develops primarily in livers affected by hepatitis or cirrhosis (2). Due to extensive tumor fibrosis, radiologists often misclassify this type of tumor as intrahepatic cholangiocarcinoma or a mixed form of hepatocellular cholangiocarcinoma (1, 3, 4). Although S-HCC has a hepatocellular structure, more than half of tumors show abundant and diffuse intratumoral fibrosis (5). Thus, this tumor type can resemble some metastatic adenocarcinomas, particularly those that are well to moderately differentiated (6). This makes it difficult for pathologists to correctly identify this uncommon tumor type.

Due to the rarity of S-HCC, treatment recommendations have not yet been established. Here, we report a case of S-HCC with response to immune checkpoint inhibitor (ICI) and vascular endothelial growth factor (VEGF) inhibitor.

## Case presentation

**Figure 1:**
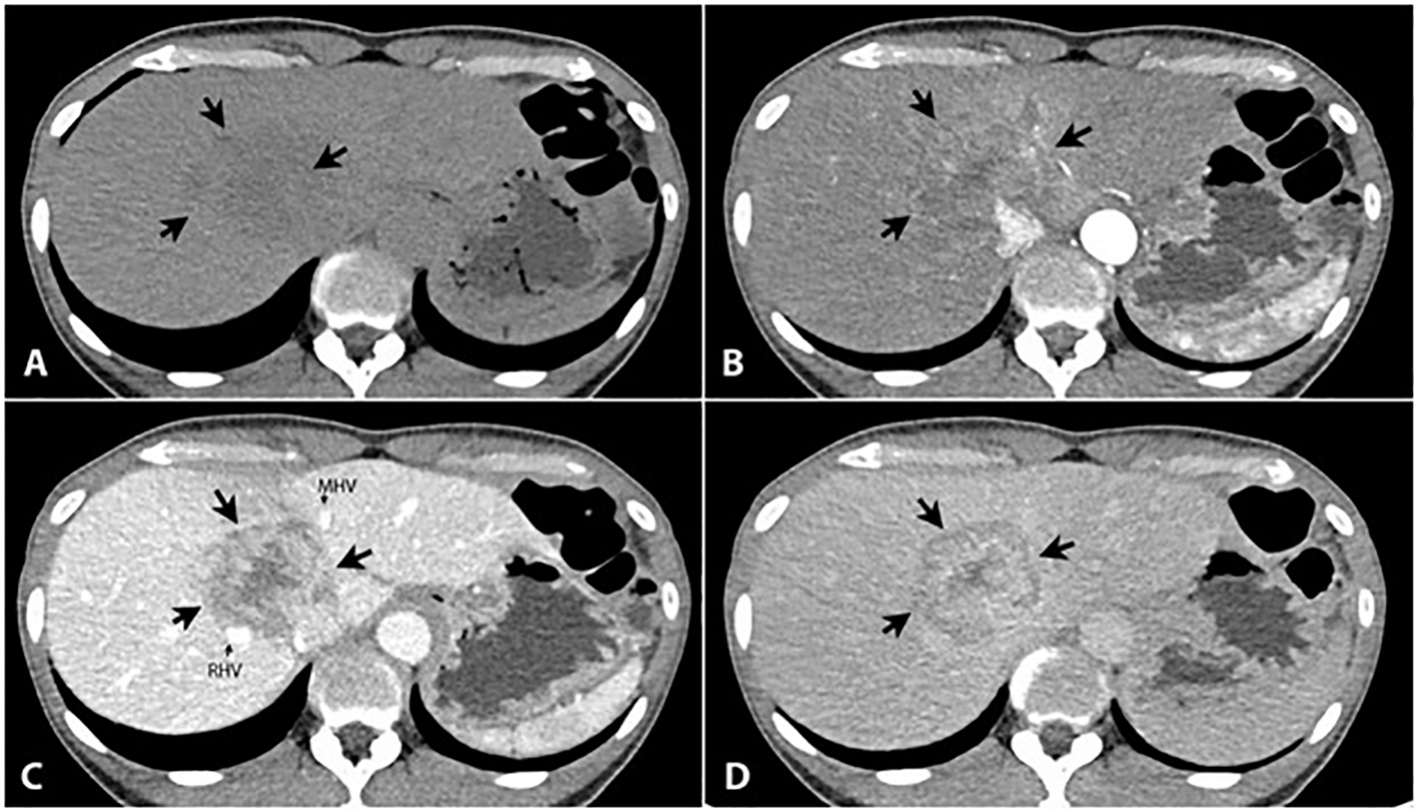
Triple phase CT of the liver reveals a large liver mass (5.6x4.6 cm) in the hepatic segment 8/4a [large arrows in **(A-D)**], presented as an ill-defined hypodense area in the precontrast phase **(A)**, heterogeneous peripheral enhancement in the arterial phase **(B)** with progressively centripetal enhancement in the portal **(C)** and 5-min delayed phases **(D)**. It shows persisted central enhancement with peripheral washout sign on 5-min delayed phase **(D)**. It adheres to the right hepatic vein (RHV, small arrow) and the middle hepatic vein (MHV, small arrow) without tumor thrombus. Several small lesions with the same appearance are scattered in both hepatic lobes (not shown).

A 50-year-old woman, without a background of cirrhosis or hepatitis B or C, presented with abdominal pain and a palpable abdominal mass. She was previously healthy. Physical examination showed palpable right lobe of liver with liver span of 13 centimeters (cm). A triple phase computed tomography (CT) of the liver revealed a 5.5 cm heterogenous peripheral arterial enhancing mass with centripetal enhancement in the portal vein and delayed phase in the hepatic segment 4a/8. This mass adhered to the right hepatic vein and the middle hepatic vein without tumor thrombus. There were also a few poorly defined heterogeneous hypodense nodules surrounding the mass with a size of up to 1.3 cm (**Figure 1**). The alpha-fetoprotein (AFP) was normal. The liver biopsy revealed moderately to poorly differentiated HCC. This was evaluated as potentially resectable HCC and as stage B (intermediate stage) on the Barcelona Clinic Liver Cancer (BCLC) staging system. Subsequently, the patient underwent local treatment, including drug-eluting beads transarterial chemoembolization with bevacizumab and embolization of the right portal vein. An extended right hepatectomy was initially planned but was abandoned due to disease progression.

**Figure 2:**
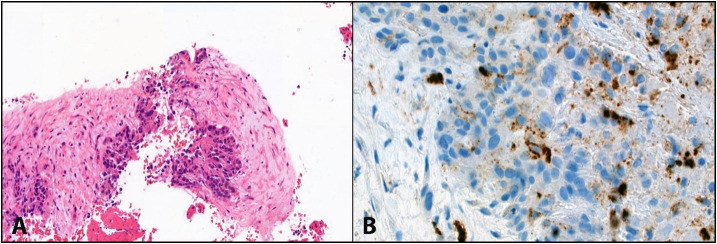
**(A)** shows abundant intratumoural fibrous stroma in scirrhous HCC. **(B)** Shows hepatocellular differentiation (CEA canalicular pattern) with fibrotic to sclerotic stroma in the surroundings, consistent with the scirrhous variant of hepatocellular carcinoma.

A triple-phase CT of the liver revealed an increase in the size of the liver mass at S4a/8 to 6 cm with multiple new hepatic lesions scattered in both hepatic lobes. A second biopsy was performed to confirm the diagnosis and showed fibrotic to sclerotic stroma (**Figure 2**). These tumor cells demonstrated hepatocellular differentiation (**Figure 2**) and strong staining with Hep Par 1, Arginase 1, CK7, and CK19, along with a canalicular pattern for CEA expression. Based on the morphology and immunohistochemical (IHC) findings, the tumor was eventually identified as scirrhous HCC, grade II variant (by Edmondson–Steiner grading).

Given her ECOG performance status of 1 and normal hepatic function, 1200 mg atezolizumab (an anti-programmed cell death ligand-1 inhibitor) combined with 700 mg bevacizumab (a VEGF inhibitor, 15 mg per kg) was administered intravenously every three weeks. Interval CT scan after 2 months of treatment showed decrease in size of tumor at segment 4a/8 from 5.5 cm to 5.1 cm The treatment was continued with good tolerance and the best response was a stable disease according to the Response Evaluation Criteria in Solid Tumors (RECIST) version 1.1 guideline. Unfortunately, after the eleventh cycle, the patient experienced weight loss, polydipsia, and polyuria and fasting blood sugar level was 360 mg/dL. Anti-Glutamic acid decarboxylase antibodies (Anti-GAD) was negative. The diagnosis of ICI-related diabetes mellitus type 1 (DM type I) was made, and this was well-controlled with four units of daily subcutaneous insulin.

**Figure 3:**
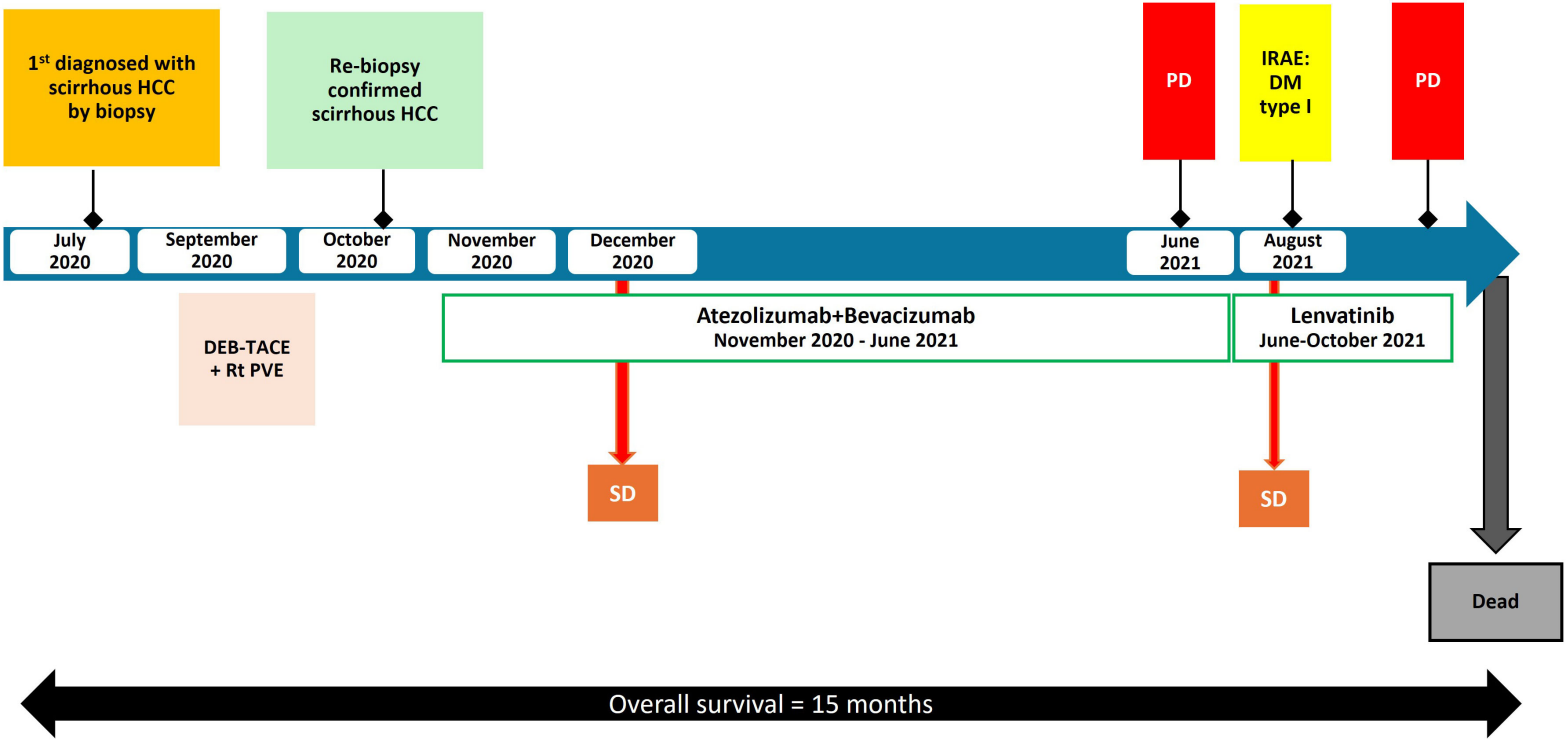
Timeline of the patient’s clinical course with key events, complications, and treatment changes. HCC, hepatocellular carcinoma; irAE, immune-related adverse event; DEB-TACE, drug-eluting bead transarterial chemoembolization; Rt PVE, right portal vein embolization; PD, progression of disease; SD, stable disease; DM, diabetes mellitus.

After 8 months of first line treatment, the patient’s abdominal pain worsened, and a CT scan revealed increase in size of liver segment 4a/8 from 5.1 cm to 8.6 cm with a new lesion in segment 5/6 (4 cm) which was considered as progressive disease (PD). Lenvatinib, a VEGF receptor inhibitor, of 8 mg/day was administered, according to the body weight of 46 kilograms as a second-line therapy. She continued on treatment without any dosing modifications. Interval CT after 2 months revealed stable disease. Unfortunately, after 4 months of lenvatinib, the patient developed obstructive jaundice due to tumor progression in liver. The patient was subsequently transitioned to best supportive care. She passed away 15 months after the initial diagnosis. **Figure 3** is a summary of the patient’s clinical timeline.

## Discussion

Recent pool data (7) indicated that S-HCC occurs frequently in patients with chronic hepatitis (60% hepatitis B, 21% hepatitis C, 16% NASH) with a mean age of 55 years; 66% of patients had elevated serum AFP levels above 20 IU/ml. Most of the liver lesions appeared as hypoechoic (48%) or mosaic patterns (48%) on ultrasound, 87% showed persistent hyperdensity in the delayed phase, 70% exhibited subcapsular liver retraction on CT scan, and 65% exhibited a target appearance on magnetic resonance imaging. These imaging findings are often misdiagnosed radiologically as intrahepatic cholangiocarcinoma or liver metastasis. The histological structure of S-HCC typically exhibited bulging appearance (100%), septation (85%), and a central scar (63%). Central necrosis is absent in about 75% of the cases. Unlike classic HCC, the IHC features of this particular variant of HCC add to the diagnostic complexity. The IHC analysis indicated that 65% were positive for Hep Par 1, 41% for CK7, and 42% for EMA while CK19 was positive only in 16% (7).

Murtha-Lemekhova et al. (8) also reported that S-HCC often exhibits higher level of CK7 and lower expression of Hep-Par1 than conventional HCC. Approximately 80% of S-HCCs are observed to exhibit glypican-3. Among these markers, CK7 and CK19 can be used to separate S-HCC from classic HCC, while Hep Par 1 can help differentiate from cholangiocarcinoma. Studies have shown that CD68 is frequently positive in fibrolamellar carcinoma, while it is typically negative in scirrhous carcinoma, indicating that it is another distinguishing marker (9). Our case report described an atypical presentation of S-HCC that originates in the liver but is not related to hepatitis or cirrhosis, and displays uncommon radiological and histopathological characteristics. In this case, the IHC revealed that the tumor was positive for both Hep Par 1 and CK7 but negative for CK20, which differs from classic HCCs that are Hep Par 1 positive but negative for both CK7 and CK20.

Due to the rarity of this subtype, the clinical outcomes of patients with S-HCC remain unknown. However, because of their aggressive characteristics, thus, treatment outcomes tend to be less favorable than those of other HCC subtypes. S-HCC has a significantly higher risk of recurrence in the first 2 years after curative therapy compared to people with non-scirrhous HCC (10) with a 5-year survival rate of 45% (6, 7). Chen XY et al. reported that the median cancer-specific survival times of the classic HCC and rare S-HCC were comparable at 15 and 13 months, respectively (11).

Characterization of the vast, thick fibrous stroma in S-HCC is thought to contribute to its resistance to therapies (12). The tumor microenvironment in S-HCC may dampen effective immune responses. As suggested by Sangro et al., it is conceivable that this dense stroma could act as a barrier, preventing immune cells from accessing the tumor (12). However, the fibrotic nature of S-HCC can indicate an inflammatory environment, making it a potential candidate for immunotherapy treatment. This potential barrier, combined with an immunosuppressive tumor environment, highlights the importance of research to improve immunotherapy, specifically for S-HCC.

The first-line standard of care for classic HCC is combination therapy consisting of ICI and VEGF inhibitors, such as in IMbrave150 trial. Based on a recent data analysis by Huang et al., S-HCC is thought to share several macroscopic clinical traits and prognoses with traditional HCC (7, 10, 13). Thus, the treatment regimen used for classic HCC may be successful for S-HCC, supporting the use of the IMbrave150 regimen in this patient.

As for prognostic factor of using immunotherapy in HCC, the MOUSEION-06 study demonstrated ECOG performance status (PS) was a pivotal prognostic factor associated with longer overall survival in patients treated with ICIs. According to Mollica et al. (14), the pool analyses of 60 studies of total 35,020 patients showed that ICIs reduced risk of death or progression in patients with good ECOG PS of 0 and 1. Up to the present, there are no validated predictive biomarkers to guide treatment selection in the HCC. Although biomarkers like PD-L1 expression, tumor Mutational Burden (TMB), and microsatellite Instability (MSI) have been useful in predicting the response to immunotherapy in other cancers, they are not yet validated as predictive biomarkers for selecting treatment in HCC.

To our knowledge, this is the first case report of S-HCC which the disease was controlled with atezolizumab and bevacizumab. In this case, the disease was stable for 8 months, which was as reported by the IMbrave 150 study. Interestingly, this case had no history of chronic hepatitis or cirrhosis background which was represented more commonly in this subtype. Her survival was 15 months, which is in line with the literature that describes a 13-month survival (11).

Currently, patients with atypical liver lesions should undergo an adequate evaluation and be referred to tertiary hospital centers for multidisciplinary team assessment and treatment. While the OS rate for patients with S-HCC is less favorable than that of those with classic HCC, this highlights the importance of tailored future treatment approaches for this aggressive subtype.

## Conclusions

Due to its often-late identification and aggressive behavior, treatment options for S-HCC are limited. Our case study demonstrated some responses to ICI and VEGF inhibitors. A better understanding of how S-HCC responds to immunotherapy requires further investigation in order to identify its distinctive characteristics and develop more effective therapeutic approaches.

## Data availability statement

The original contributions presented in the study are included in the article/supplementary material. Further inquiries can be directed to the corresponding author.

## Ethics statement

Written informed consent was obtained from the individual(s) for the publication of any potentially identifiable images or data included in this article.

## Author contributions

AA: Conceptualization, Visualization, Writing – original draft, Writing – review & editing. NK: Resources, Visualization, Writing – review & editing. SS: Visualization, Writing – review & editing. PA: Resources, Visualization, Writing – review & editing. CA: Visualization, Writing – review & editing. KK: Resources, Supervision, Visualization, Writing – review & editing, Conceptualization.
